# Variations in agronomic and grain quality traits of rice grown under irrigated lowland conditions in West Africa

**DOI:** 10.1002/fsn3.635

**Published:** 2018-04-02

**Authors:** Seth Graham‐Acquaah, Kazuki Saito, Karim Traore, Ibnou Dieng, Amakoe Alognon, Saidu Bah, Abdoulaye Sow, John T. Manful

**Affiliations:** ^1^ Africa Rice Center (AfricaRice) Cotonou Benin; ^2^ Africa Rice Center (AfricaRice) Bouaké Côte d'Ivoire; ^3^ Africa Rice Center (AfricaRice) Saint‐Louis Senegal; ^4^ Present address: Department of Food Science University of Arkansas Fayetteville AR USA; ^5^ Present address: Ministry of Food and Agriculture Accra Ghana

**Keywords:** breeding, effective parameters, heterogeneity, rice

## Abstract

Rice breeding in West Africa has been largely skewed toward yield enhancement and stress tolerance. This has led to the variable grain quality of locally produced rice in the region. This study sought to assess variations in the agronomic and grain quality traits of some rice varieties grown in this region, with a view to identifying sources of high grain yield and quality that could serve as potential donors in their breeding programs. Forty‐five varieties were grown under irrigated conditions in Benin and Senegal with two trials in each country. There were wide variations in agronomic and grain quality traits among the varieties across the trials. Cluster analysis using paddy yield, head rice yield, and chalkiness revealed that 68% of the total variation could be explained by five varietal groupings. One group comprising seven varieties (Afrihikari, BG90‐2, IR64, Sahel 108, WAT311‐WAS‐B‐B‐23‐7‐1, WAT339‐TGR‐5‐2, and WITA 10) had high head rice yield and low chalkiness. Of the varieties in this group, Sahel 108 had the highest paddy yield in three of the four trials. IR64 and Afrihikari had intermediate and low amylose content, respectively, with the rest being high‐amylose varieties. Another group of varieties consisting of B6144F‐MR‐6‐0‐0, C74, IR31851‐96‐2‐3‐2‐1, ITA222, Jaya, Sahel 305, WITA 1, and WITA 2 had high paddy yield but poor head rice yield and chalkiness. The use of materials from these two groups of varieties could accelerate breeding for high yielding rice varieties with better grain quality for local production in West Africa.

## 1 INTRODUCTION

Rice (*Oryza* spp.) is an important staple in many parts of the world. In West Africa, the increasing demand for rice surpasses local production. Thus, many countries rely on imports to meet consumer demands. To meet the production deficit, rice breeding programs in this region have focused mainly on yield enhancement and adaptation to the harsh production environments than on improving grain quality (Manful, 2010). Consequently, yield and stress tolerance have improved (Saito, Sokei, & Wopereis, 2012) but issues of quality continue to plague local production efforts. The lower quality of locally produced rice available in the market is due to the combined effects of the types of varieties grown, poor postharvest management, and processing practices (Futakuchi, Manful, & Sakurai, 2013). Most locally cultivated varieties have low milling recoveries, high incidence of chalkiness, and poor cooking characteristics (Africa Rice Center, 2011), resulting in discounted prices of locally produced *vis‐a‐vis* imported rice.

As consumers’ incomes increase and markets become more liberalized, consumers’ preferences for rice have been shown to shift from lower to higher quality (Cuevas & Fitzgerald, 2012). Likewise, as both the populations and earnings of urban settlers in West Africa continue to grow, the demand for high‐quality rice is increasing (Demont & Ndour, 2015). This situation requires breeding programs to develop varieties that can match the quality of imported rice being sold on urban markets. The crossing of parental lines known to possess desirable grain qualities with high yielding lines is a first step toward enhancing both traits through breeding. However, the quality characteristics of rice varieties in West Africa, including those widely used as parental lines in breeding programs in the region, are not well documented.

Rice grain quality is a composite of several characteristics—appearance, milling, cooking, and eating characteristics. Unlike preferences for cooking and eating quality, which vary markedly from region to region (Calingacion et al., 2014; Champagne et al., 2010), the requirements for milling recovery and appearance are universal; a high grain quality variety should have high head rice yield after milling and should have low incidence of chalkiness (Fitzgerald, McCouch, & Hall, 2009; Lyman, Jagadish, Nalley, Dixon, & Siebenmorgen, 2013; Nelson et al., 2012). Rice varieties differ in their grain quality traits and more so in their head rice yield and chalkiness (Anacleto et al., 2015; Koutroubas, Mazzini, Pons, & Ntanos, 2004; Tong et al., 2014). Not only are there marked differences in rice quality among genotypes but also growing environment has been shown to affect rice appearance, milling, and eating quality (Ashida, Araki, Maruyama‐Funatsuki, Fujimoto, & Ikegami, 2013; Bao, Kong, Xie, & Xu, 2004; Champagne, Bett‐Garber, McClung, & Bergman, 2004; Liu, Wu, Ma, & Xin, 2015). Among grain quality traits, head rice yield and chalkiness are the two that have been observed to be most significantly affected by environment (Zhao & Fitzgerald, 2013). Using cultivars with consistently high grain quality indices as parents in reducing quality variation is a way of maintaining the reputation of varieties. However, little is documented on the performance of parental lines used in breeding programs as well as locally grown varieties in West Africa with respect to their grain quality characteristics across different environments.

The objectives of this study were therefore to: (1) assess varietal difference in agronomic traits including rice paddy yield and grain quality characteristics; (2) quantify the effects of environment and genotype by environment interaction on them; and (3) identify varieties that combine high paddy yield, high head rice yield, and low chalkiness that could be exploited in breeding programs.

## 2 MATERIALS AND METHODS

### 2.1 Varieties

The following 45 rice varieties obtained from the Genetic Resources Unit of the Africa Rice Center (AfricaRice) were evaluated in this study: BG90‐2, C74, IR1529‐680‐3, IR31785‐58‐1‐2‐3‐3, IR31851‐96‐2‐3‐2‐1, IR64, Jaya, Kogoni 91‐1, Sahel 108, Sahel 177, Sahel 201, Sahel 217, Sahel 222, Sahel 305, Sahel 328, Sahel 329, and WAS161‐B‐9‐3; Afrihikari, Bouake 189, FKR19 (Tox728‐1), FKR54 (WABIR12979), NERICA‐L 19, WAT100‐TGR‐2‐4, WAT307‐WAS‐B‐24‐8‐4‐4‐2, WAT311‐WAS‐B‐B‐23‐7‐1, WAT317‐WAS‐B‐B‐55‐4‐3, WAT339‐TGR‐5‐2, WAT343‐TGR‐1‐1, WAT50‐TGR‐4‐1, WITA 1, WITA 2, WITA 3, WITA 4, WITA 6, WITA 7, WITA 10 and WITA 12, three mangrove varieties—WAR42‐82‐2‐3‐1, WAR77‐3‐2‐2, and WAR87‐10‐2‐2‐9; and B6144F‐MR‐6‐0‐0, ITA123, ITA222, WAB337‐B‐B‐7‐H4, and WAB506‐125‐3.

### 2.2 Experimental design and trial setup

Four experiments were conducted under irrigated lowland conditions in Benin and Senegal with two trials per country. In Benin, the trials were conducted during the same cropping season (August to December) in both 2011 and 2012 at the AfricaRice experimental station in Cotonou (6°25′N, 2°20′E). In Senegal, the first trial was established in February 2012 (dry season) and the second trial in July 2012 (wet season) at the AfricaRice experimental station, Ndiaye (16°14′N, 15°13′W). Cotonou is located in the subhumid zone, whereas Ndiaye is located in the arid zone (Saito et al., 2013). The Senegal site has 200 mm average annual rainfall with one rainy season (July–September), a high solar radiation of 20–30 MJ/(m^2^ day) for most of the year, and higher temperature amplitudes. In comparison, the Benin site has higher annual rainfall and humidity, and lower solar radiation and temperature amplitudes (Saito & Futakuchi, 2009). Potential paddy yield, determined by crop simulation model, is generally higher in arid zone than in subhumid zones (Becker, Johnson, Wopereis, & Sow, 2003; van Oort et al., 2015).

An alpha lattice design was used with two replications in Benin and three replications in Senegal. Plot size was 2.6 m × 2 m. Rice seedlings were transplanted at 18–21 days after sowing at a density of 20 cm × 20 cm with one plant per hill. Fertilizer was applied at 200 kg/ha of compound NPK fertilizer (15‐15‐15 or 10‐20‐20) at transplanting time and 40 kg N/ha as urea at 4 weeks after transplanting. Pests and diseases were controlled as and when required. Weeds were manually removed, when required.

The number of days to heading was recorded in each plot. Plant height (from the soil surface to the tip of the panicle of the tallest plant, excluding the awns) was measured at maturity on five randomly selected hills. Twelve plants were then randomly chosen from each plot and harvested to determine the number of panicles, number of grains per panicle, and paddy grain yield. Paddy yield was adjusted to 14% moisture content.

### 2.3 Grain quality evaluation

After harvesting 12 hills, the remaining plants less one border row was used for grain quality evaluation. Harvested paddy was dried in the sun to a moisture content of 18% and then dried in the shade to between 12% and 14% moisture content. The dried samples were kept in paper bags and equilibrated at laboratory temperature for 1 month prior to grain quality evaluation.

#### 2.3.1 Milling recoveries

Rice samples were dehusked in a THU‐34A Satake Testing Rice Husker (Satake, Japan). The brown rice obtained was polished in a Ricepal 32 (Yamamoto Co., Japan) rice polisher. Milled rice was separated into whole and broken grains using a Satake Test Rice Grader (Satake, Japan). Head rice yield was calculated using the following equations:

(1) $$\text{Brown Rice Yield}\;\left(\%\right)=\frac{\text{weight of dehusked rice}}{\text{weight of paddy}}\times 100$$

(2) $$\text{Total Milling Yield}\;\left(\%\right)=\frac{\text{weight of milled rice}}{\text{weight of paddy}}\times 100$$

(3) $$\text{Head Rice Yield}\;\left(\%\right)=\frac{\text{weight of whole grains}}{\text{weight of paddy}}\times 100$$

#### 2.3.2 Chalkiness and grain dimensions

Chalkiness and grain dimensions were determined using S21 Rice Statistic Analyzer (LKL Technologia, Brazil) as described by Graham‐Acquaah, Manful, Ndindeng, and Tchatcha (2015) with slight modifications. Chalkiness was determined by processing the captured images and applying the “basic filter—chalky distribution.” The percentage of total chalky area for the samples were recorded and reported as the percentage chalkiness of the samples. The grain dimensions were determined by applying the “advanced filter‐length distribution” on the software. The grain length and width were then recorded, and the length/width ratio was calculated.

#### 2.3.3 Apparent amylose content

Apparent amylose content was measured using the standard iodine colorimetric method ISO 6647‐2‐2011. Absorbance of the solution was measured using an AutoAnalyzer 3 (Seal Analytical, Germany) at 600 nm, and apparent amylose content was quantified from a standard curve generated from absorbance values of four well‐known standard rice varieties (IR65, IR24, IR64, and IR8).

#### 2.3.4 Pasting properties

The pasting properties of rice flour samples were measured using a Rapid Visco‐Analyzer (RVA) model super4 (Newport Scientific, Warriewood, Australia) and Thermocline for Windows (TCW3) software. The general pasting method 162 (ICC, 2004) for flour samples was used.

### 2.4 Data analyses

Analysis of variance using linear mixed models regression (REML) analysis was run in “R” version 3.2.2 (R Core Team, 2015) to determine the effects of variety (genotype), trial (environment), and genotype x environment interaction on the measured traits. Pearson's correlation coefficients in each trial were calculated based on predicted means generated from REML analyses. Hierarchical cluster analysis was carried out using the mean values of paddy yield, head rice yield, and chalkiness across the four trials. Values of traits were transformed to Z scores where necessary to facilitate their visualization on the same scale.

## 3 RESULTS

### 3.1 Varietal and environmental variations in agronomic and grain quality characteristics of rice varieties

Analyses of variance showed that the main effects of both genotype and environment, and genotype x environment interaction were significant (*p* < .05) for all the agronomic and grain quality traits except for the interaction on apparent amylose content and grain shape, given by the length‐to‐width ratio (Table 1). Average paddy yield across the 45 varieties was higher in the two trials in Senegal than in those in Benin and higher in the dry season than in the wet season trial in Senegal (Table 2). Averaged over the 45 varieties, head rice yield was higher in Senegal, especially in the dry season. The average percentage grain chalkiness was higher in the wet season in Senegal than in the other three trials. Compared with head rice and chalkiness, apparent amylose content and paste properties of varieties were influenced to a lesser extent by the environment.

**Table 1:** Analysis of variance and heritability of agronomic and grain quality traits of 45 rice varieties grown under irrigated lowland conditions in Benin and Senegal across environments

Factors	Agronomic traits						Grain quality traits										
	Days to heading	Plant height (cm)	Number of panicles	Grains per panicle	1,000‐Grain weight (g)	Grain yield (g)	Brown rice yield (%)	Total milling yield (%)	Head rice yield (%)	Chalkiness (%)	Apparent amylose content (%)	Grain length (mm)	Grain width (mm)	Length to width ratio	Peak viscosity (cP)	Breakdown viscosity (cP)	Setback viscosity (cP)
Variety (G)	2,231.1***	3,012.8***	317.7***	225.3***	672.9***	231.6***	187.3***	153.4***	291.8***	237.5***	577.8***	47.9***	19.8***	50.8***	1,636.7***	1,261.3***	1,530.4***
Environment (E)	570.4***	91.0***	145.1.2***	24.0***	11.44**	27.0***	13.6**	13.3**	32.3***	64.0***	1	33.0***	16.7***	31.8***	65.3***	54.9***	83.6***
G×E	3,042.0***	357.5***	349.9***	181.8**	278.2***	380.5***	245.5***	231.7***	187.7**	221.6***	57.6	5.6*	1.0**	2.3	292.0***	332.5***	383.4***
Heritability																	
Benin 2011	93	94	84	75	89	48	39	11	41	39	‐	85	85	88	87	88	90
Benin 2012	92	89	68	56	56	53	8	75	66	16	‐	83	96	96	87	93	97
Senegal dry season	99	96	80	61	91	88	77	82	86	74	89	85	95	92	96	94	95
Senegal wet season	80	97	81	48	82	61	74	62	59	74	85	62	80	74	93	87	88

HDDAYS, days to heading; PLNTHT, plant height in cm; PNCLNBR, number of panicles; GRNSPNCL, number of grains per panicle; 1,000‐GRNWT, weight of 1,000 grains; GRNYIELD, paddy yield; BRY, brown rice yield; TMY, total milling yield; HRY, head rice yield; AAC, apparent amylose content; GRNLNT, grain length; GRNWDT, grain width; LWR, length‐to‐width ratio; PkV, peak viscosity; BD, breakdown viscosity; SB, setback viscosity.

***significant at *p* < .001; ** significant at *p* < .01; *significant at *p* < .05; G*E analysis was conducted using only data from Senegal.

**Table 2:** Minimum, maximum, and means of agronomic and grain quality characteristics of 45 rice varieties grown under irrigated lowland conditions in Benin and Senegal

Trait	2011 in Cotonou			2012 in Cotonou			Dry season in Senegal			Wet season in Senegal		
	Min	Max	Mean	Min	Max	Mean	Min	Max	Mean	Min	Max	Mean
Agronomic traits												
Days to heading	88	105	97	74	101	86	101	233	132	76	112	99
Plant height (cm)	60	141	86	84	143	105	89	196	117	71	150	98
Number of panicles	6	14	8	5	12	8	4	22	14	7	30	17
Grains per panicle	86	200	144	72	239	127	56	168	119	52	157	87
1,000‐grain weight (g)	23	35	28	24	33	28	22	34	27	23	38	28
Grain yield (g)	364	981	625	329	706	538	57	1,199	871	398	1,040	749
Grain quality traits												
Brown rice yield (%)	76	82	79	76	81	78	72	82	80	76	82	80
Total milling yield (%)	49	70	64	62	71	66	52	72	66	53	69	64
Head rice yield (%)	8	62	38	6	51	30	19	66	48	15	60	40
Chalkiness (%)	3	44	19	9	34	19	9	34	21	9	50	27
Apparent amylose content (%)	12	29	26	11	32	28	13	31	27	13	30	26
Grain length (mm)	5.1	6.9	6.4	5	6.8	6.3	5	6.7	6.3	4.9	6.9	6.3
Grain width (mm)	1.9	2.6	2.2	1.9	2.6	2.2	1.9	2.6	2.2	1.8	2.4	2.1
Length‐to‐width ratio	2.1	3.4	3	2	3.3	3	2.2	3.6	3	2.6	3.5	3.1
Peak viscosity (cP)	1,442	3,401	2,464	1,575	3,924	2,895	1,532	3,742	2,819	1,259	3,700	2,500
Breakdown viscosity (cP)	244	1,392	721	218	1,627	872	276	1,714	950	108	1,712	664
Setback viscosity (cP)	−347	2,054	1,053	−512	1,865	889	−539	2,035	821	−400	2,016	1,087

As most of the traits had significant genotype x environment interaction, results obtained from each trial were analyzed separately. Heritability of paddy yield, brown rice yield, total milling yield, head rice yield, and chalkiness varied among the four trials, and these traits generally had lower heritability than amylose content, grain dimensions, and paste viscosities (Table 1).

Paddy yield (g/m^2^) ranged from 364 to 981 (Benin 2011), 329 to 701 (Benin 2012), 57 to 1,199 (Senegal 2012 dry season), and 398 to 1,040 (Senegal 2012 wet season). Similarly, the number of days to heading ranged from 88 to 105 (Benin 2011), 74 to 101 (Benin 2012), 101 to 233 (Senegal 2012 dry season), and 76 to 112 (Senegal 2012 wet season). The largest ranges in yield and days to heading were observed in the dry season in Senegal because some of the varieties were not adapted to dry season conditions, especially the longer day length in that season. There were large varietal differences in head rice yield than total milling yield. Head rice yields (%) ranged from 8 to 62 (Benin 2011), 6 to 51 (Benin 2012), 19 to 66 (Senegal 2012 dry season), and 15 to 60 (Senegal 2012 wet season). The ranges for grain chalkiness, grain dimensions (grain length, grain width, and length‐to‐width ratio), apparent amylose content, peak viscosity, and setback viscosity were similar across the four trials. The apparent amylose contents of the varieties in this study covered the range of low‐ to high‐amylose content. None of the varieties tested was waxy or had very low amylose content (<10% AAC).

**Figure 1:**
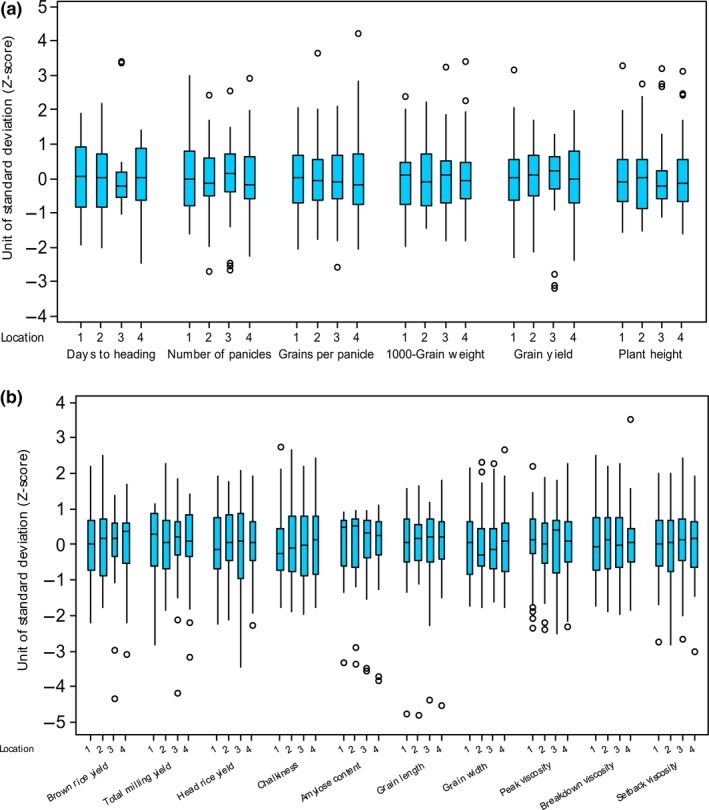
Variations in (a) agronomic and (b) grain quality traits of 45 rice varieties grown under irrigated conditions in Benin and Senegal

Figure 1 illustrates the apparent diversity in the agronomic and grain quality traits of the 45 varieties. Across the four trials, differences among the varieties were higher for milling recoveries (in particular head rice yield), chalkiness, and pasting properties than for other agronomic and grain quality traits; the reverse was the case for amylose content and grain dimensions. Differences in varietal traits, such as days to heading, plant height, and paddy yield, were relatively lower in the dry season in Senegal (Figure 1), despite the large ranges in their minimum and maximum values (Table 2); this is because several varieties were outliers in those traits.

### 3.2 Relationships among agronomic and grain quality traits

Pearson's correlation coefficients were computed to ascertain pairwise relationships among the traits for each trial. Table 3 lists the pairs of traits having significant and consistent correlations (*p* < .05) across the four trials. Paddy yield and number of panicles had positive correlations (*r* = .48–.77). None of the agronomic traits was correlated consistently with any of the grain quality traits. Among the grain quality traits, there were positive correlations between brown rice yield and total milling yield, and between total milling yield and head rice yield. Apparent amylose content was negatively correlated with head rice yield, grain width, and breakdown viscosity, whereas it was positively correlated with length‐to‐width ratio and setback viscosity. Peak viscosity had a positive correlation with breakdown viscosity and a negative correlation with setback viscosity.

**Table 3:** Correlations among agronomic and grain quality traits across trials

Traits	Range of correlations	Level of significance			
		2011 in Cotonou	2012 in Cotonou	Senegal dry season	Senegal wet season
Grain yield vs. number of panicles	0.48 to 0.77	***	***	***	***
Brown rice yield vs. total milling yield	0.29 to 0.68	*	*	***	***
Total milling yield vs. head rice yield	0.52 to 0.76	***	***	***	***
Head rice yield vs. apparent amylose content	−0.30 to −0.56	*	**	**	***
Grain length vs. length width ratio	0.50 to 0.70	***	***	***	***
Grain width vs. length width ratio	−0.84 to −0.91	***	***	***	***
Grain width vs. apparent amylose content	−0.35 to −0.44	*	*	**	**
Length width ratio vs. apparent amylose content	0.35 to 0.46	**	**	**	*
Apparent amylose content vs. breakdown viscosity	−0.52 to −0.62	***	***	***	***
Apparent amylose content vs. setback viscosity	0.65 to 0.70	***	***	***	***
Peak viscosity vs. breakdown viscosity	0.47 to 0.63	***	***	***	**
Breakdown viscosity vs. setback viscosity	−0.65 to −0.86	***	***	***	***

***significant at *p* < .001; **significant at *p* < .01; *significant at *p* < .05.

### 3.3 Classification of varieties based on paddy yield, head rice yield, and chalkiness across trials

**Figure 2:**
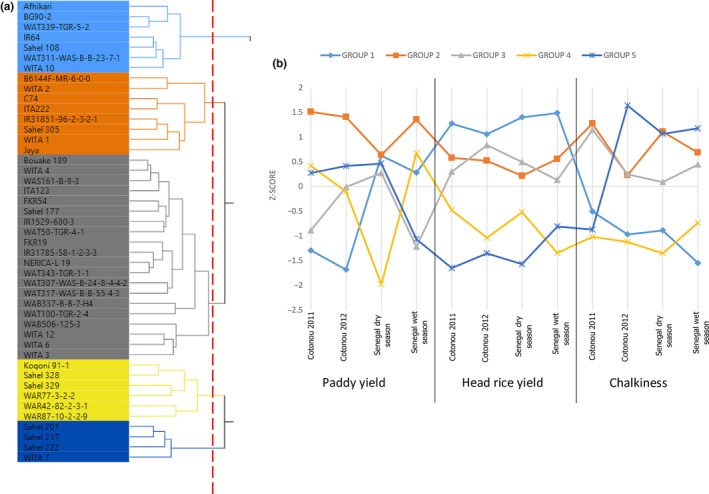
(a) Clusters of varieties based on the paddy yield, head rice yield, and chalkiness. (b) Paddy yield, head, and chalkiness performance of groups of rice varieties across trials

Ward's hierarchical cluster analyses on paddy yield, head rice yield, and chalkiness across the four trials identified five variety clusters (referred to as Clusters 1–5) that could explain 68% of total variation (Figure 2). Cluster 1 comprised seven varieties—Sahel 108, WITA 10, IR64, Afrihikari, BG90‐2, WAT339‐TGR‐5‐2, and WAT311‐WAS‐B‐B‐23‐7‐1 (Table 4). This cluster is characterized by desirable grain quality (high head rice recovery and low chalkiness) that is stable across the four trials and relatively poor adaptation to Benin in terms of paddy yield. Sahel 108 had the highest paddy yield within this cluster in all the trials except for the dry season trial in Senegal where it had the second highest yield after WAT311‐WAS‐B‐B‐23‐7‐1.

**Table 4:** Agronomic and grain quality traits of rice varieties in Cluster 1

Variety	Days to heading	Plant height (cm)	Number of panicles	Grains per panicle	1,000‐grain weight (g)	Grain yield (g)	Brown rice yield (%)	Total milling yield (%)	Head rice yield (%)	Chalkiness (%)	Apparent amylose content (%)	Grain length (mm)	Grain width (mm)	Length to width ratio	Peak viscosity (cP)	Breakdown viscosity (cP)	Setback viscosity (cP)
Cotonou 2011																	
Afrihikari	89	66	7	86	25	364	80	70	62	17	12	5.1	2.4	2.1	2,664	1,054	183
BG90‐2	103	70	10	100	26	552	77	58	34	16	28	6.1	2.1	2.9	2,768	415	1,705
IR64	90	60	9	93	29	600	79	68	55	17	23	6.7	2.3	3.0	2,648	1,053	376
Sahel 108	93	72	11	124	26	677	79	69	53	9	28	6.2	2.0	3.1	1,725	430	1,127
WAT311‐WAS‐B‐B‐23‐7‐1	88	69	8	118	29	551	80	70	43	20	28	6.6	2.1	3.2	2,377	606	1,381
WAT339‐TGR‐5‐2	96	87	9	119	24	484	78	66	49	16	28	6.3	2.0	3.1	2,388	509	1,691
WITA 10	103	76	11	144	23	577	81	69	62	8	26	6.2	2.1	3.0	2,435	262	2,054
Cotonou 2012																	
Afrihikari	77	85	8	87	26	329	81	71	41	18	14	5.0	2.5	2.0	3,035	1,231	55
BG90‐2	91	94	10	80	26	451	77	66	29	14	30	6.1	2.1	2.9	3,016	440	1,748
IR64	81	92	9	96	28	485	79	67	38	16	22	6.3	2.1	3.1	2,659	1,013	509
Sahel 108	78	85	9	129	25	603	79	64	43	14	31	6.4	2.0	3.2	2,254	631	994
WAT311‐WAS‐B‐B‐23‐7‐1	74	87	8	126	29	497	79	66	35	20	31	6.6	2.0	3.2	2,669	724	1,197
WAT339‐TGR‐5‐2	82	107	8	109	30	400	79	64	31	13	30	6.2	1.9	3.3	2,547	360	1,865
WITA 10	84	84	7	94	29	436	79	66	41	26	32	6.4	2.2	2.9	2,524	642	1,220
Senegal dry season																	
Afrihikari	105	102	16	108	22	865	79	70	63	15	13	4.9	2.6	2.2	3,151	1,507	−212
BG90‐2	143	102	16	97	26	1,002	79	69	58	17	29	6.0	2.2	2.8	2,754	497	1,365
IR64	121	99	12	129	27	871	81	68	55	17	24	6.4	2.1	3.1	3,007	1,431	120
Sahel 108	114	91	15	168	22	1,097	80	66	55	15	30	6.1	2.0	3.1	2,409	856	1,012
WAT311‐WAS‐B‐B‐23‐7‐1	111	96	19	106	27	1,199	79	67	55	16	26	6.4	2.0	3.2	2,696	883	1,074
WAT339‐TGR‐5‐2	123	119	15	104	22	757	80	69	57	15	29	6.2	2.0	3.1	2,472	761	1,192
WITA 10	138	107	18	105	25	1,055	80	67	62	15	29	6.1	2.2	2.9	3,282	460	1,507
Senegal wet season																	
Afrihikari	79	80	16	57	27	544	79	66	57	16	13	4.9	2.3	2.6	2,747	1,133	366
BG90‐2	108	84	16	75	27	759	77	65	48	13	27	6.0	2.1	3.0	2,603	293	1,676
IR64	92	92	15	112	29	827	80	68	58	13	24	6.4	2.0	3.2	2,459	1,005	541
Sahel 108	97	86	21	94	25	914	80	66	50	16	26	6.1	2.0	3.1	1,940	720	737
WAT311‐WAS‐B‐B‐23‐7‐1	92	81	23	66	27	895	80	65	46	19	28	6.4	1.9	3.3	2,239	668	1,028
WAT339‐TGR‐5‐2	97	105	15	100	25	681	79	65	47	12	29	6.3	1.9	3.3	2,916	654	1,755
WITA 10	110	96	17	105	28	912	81	68	43	11	27	6.3	2.0	3.1	2,564	108	1,924

Furthermore, of the 45 varieties, paddy yield of Sahel 108 ranked 13th (Benin 2011), 11th (Benin 2012), 5th (Senegal dry season 2012), and 5th (Senegal 2012 wet season). Sahel 108 also had the most stable head rice yield (42%–53%) among the varieties in this cluster, and, on average over the four trials, the lowest grain chalkiness and the 4th most stable chalkiness level. Afrihikari gave the highest mean head rice yield within this cluster and its head rice yield ranked 1st (Benin 2011), 9th (Benin 2012), 2nd (Senegal 2012 dry season), and 3rd (Senegal 2012 wet season) of the 45 varieties. All the varieties in this cluster, except Afrihikari (low amylose; AAC < 20%) and IR64 (intermediate amylose; AAC 20%–25%), had high‐amylose contents (AAC > 25%).

Cluster 2 comprised eight varieties—B6144F‐MR‐6‐0‐0, C74, IR31851‐96‐2‐3‐2‐1, ITA222, Jaya, Sahel 305, WITA 1, and WITA 2 (Tables 5). Generally, this group is characterized by high paddy yields, low head rice yield, and high incidence of chalkiness (Figure 2b). Jaya produced higher paddy yield than Sahel 108 in three of four trials. Cluster 3 had the largest number of varieties (20) (Figure 2), but none of the three traits of this cluster showed exceptional or consistent performance across the four trials. Paddy yield was variable across trials, whereas head rice yield and grain translucency were moderate to poor.

**Table 5:** Agronomic and grain quality traits of varieties in Cluster 2

Variety	Days to heading	Plant height (cm)	Number of panicles	Grains per panicle	1000‐grain weight (g)	Grain yield (g)	Brown rice yield (%)	Total milling yield (%)	Head rice yield (%)	Chalkiness (%)	Apparent amylose content (%)	Grain length (mm)	Grain width (mm)	Length to width ratio	Peak viscosity (cP)	Breakdown viscosity (cP)	Setback viscosity (cP)
Cotonou 2011																	
B6144F‐MR‐6‐0‐0	95	103	7	163	29	739	80	69	45	30	26	6.3	2.5	2.6	2,568	617	1,326
C74	97	104	11	128	31	856	80	66	44	16	28	6.7	2.2	3.1	2,513	600	1,378
IR31851‐96‐2‐3‐2‐1	92	70	9	143	25	654	80	69	47	12	23	6.1	2	3	2,405	916	714
ITA222	101	80	14	155	30	981	80	65	36	22	28	6.6	2.3	2.9	2,761	769	1,532
Jaya	100	85	9	173	32	741	80	69	46	37	28	6.5	2.5	2.7	3,077	441	1,936
Sahel 305	96	81	7	194	26	676	81	64	47	15	26	6.5	2	3.3	1,564	248	994
WITA 1	95	65	10	101	31	630	79	70	58	17	24	6.4	2.4	2.7	2,571	923	873
WITA 2	97	85	11	160	30	822	79	49	15	38	28	6.7	2.2	3	2,873	693	1,269
Cotonou 2012																	
B6144F‐MR‐6‐0‐0	92	122	6	134	26	451	80	70	34	30	28	6.2	2.4	2.6	3,157	798	1,119
C74	88	116	9	119	30	598	79	67	36	16	23	6.7	2.2	3.1	3,087	826	1,219
IR31851‐96‐2‐3‐2‐1	79	93	10	110	32	646	79	67	29	12	25	6.2	2.1	2.9	2,889	1,119	506
ITA222	89	102	9	139	30	629	77	65	28	21	31	6.5	2.2	2.9	3,075	741	1,280
Jaya	94	107	8	148	30	631	77	69	23	16	32	6.5	2.5	2.6	3,894	763	1,433
Sahel 305	85	105	9	239	24	700	79	63	31	19	31	6.3	1.9	3.3	1,575	218	853
WITA 1	89	101	10	189	25	627	78	67	48	13	30	6	2	2.9	2,868	223	1,732
WITA 2	91	98	8	117	29	567	80	64	26	34	28	6.3	2.1	3	3,596	1,014	934
Senegal dry season																	
B6144F‐MR‐6‐0‐0	121	138	10	155	26	853	79	68	47	28	28	5.6	2.4	2.4	3,075	933	1,170
C74	138	149	16	97	31	995	81	70	62	25	29	6.6	2.2	3	2,975	945	959
IR31851‐96‐2‐3‐2‐1	114	99	13	136	23	858	80	64	40	25	25	6.1	2	3	2,856	1,282	553
ITA222	131	99	16	120	27	1,045	81	65	50	32	30	6.4	2.2	2.9	3,214	907	1,038
Jaya	131	104	12	124	29	924	80	68	35	32	31	6.3	2.5	2.5	3,742	617	1,421
Sahel 305	117	109	12	167	25	1,070	82	66	49	18	30	6.6	1.9	3.4	1680	276	1156
WITA 1	131	103	16	102	29	1082	81	66	51	32	29	6.5	2.2	3	2,508	809	1,044
WITA 2	127	103	16	117	28	1,026	80	64	46	27	27	6.4	2.1	3	3,059	1,015	609
Senegal wet season																	
B6144F‐MR‐6‐0‐0	91	110	19	78	31	1,040	80	69	53	41	28	6	2.3	2.6	2,916	691	1,349
C74	112	113	13	85	30	659	81	67	45	30	28	6.7	2.1	3.1	2,818	592	1,553
IR31851‐96‐2‐3‐2‐1	100	83	26	83	27	901	80	64	43	27	26	6.2	2	3.1	2,726	1,114	653
ITA222	106	90	15	88	30	728	80	64	43	34	29	6.6	2.2	3	2,783	554	1,555
Jaya	99	84	30	62	32	1,008	79	63	31	35	29	6.1	2.3	2.7	3,483	358	2,016
Sahel 305	88	89	17	94	26	910	81	61	37	17	28	6.5	1.9	3.4	1,339	153	793
WITA 1	93	88	20	67	30	838	80	68	53	29	22	6	2.3	2.7	2,433	849	651
WITA 2	108	88	20	98	29	888	80	63	37	37	29	6.6	2.1	3.2	3,294	730	1,343

There were six varieties in Cluster 4 and four in Cluster 5. Both clusters had variable paddy yield and consistently low head rice yields. Cluster 4 generally had the lowest incidence of chalkiness in all the trials except for the wet season trial in Senegal.

## 4 DISCUSSION

### 4.1 Agronomic and grain quality characteristics of varieties across trials

This study found that apart from significant effects of environment and genotype by environment interaction on almost all the traits determined, there are substantial varietal differences in paddy yield and other agronomic traits that can be exploited in rice breeding programs. The results confirm reports on similarly large variations in paddy yield of rice grown under irrigated lowland conditions in West Africa (Saito, Azoma, & Sié, 2010; Saito, Azoma, & Sokei, 2010) as well as findings that the arid zone has a higher yield potential than the subhumid zone in West Africa and that potential yield is higher in dry season than in the wet season in the arid zone (Becker et al., 2003; van Oort et al., 2015).

Because grain quality has often been deemed secondary to yield as far as rice breeding programs in Africa are concerned, reports showing varietal differences in grain quality traits in this region are scanty, more so where grain quality is evaluated alongside agronomic characteristics. With the surge in urban populations in Africa and the demand of these urban settlers for rice of superior quality increases, breeding programs are now obliged to deliver varieties that can match the quality of imported rice on local markets; this study does not only show the apparent diversity in the appearance, milling, and eating characteristics of rice varieties in the region but also the potential within these local cultivars that can be exploited to breed for better quality rice in the region. The highest genotypic variability in grain quality traits across trials was observed for head rice yield, chalkiness, and pasting properties. These observed genotypic variations are enough to justify crosses between lower yielding varieties to improve milling, appearance, and eating quality of rice in the region.

Milling recoveries observed in this study are largely consistent with those of Koutroubas et al. (2004) and Liu et al. (2015). Higher head rice yields observed in the dry season compared with the other trials in this study are consistent with the findings of Zhao and Fitzgerald (2013) who observed that the average head rice yields of 39 varieties cultivated for 4 years were consistently higher during dry seasons than wet seasons. Zhou et al. (2015) also reported a 10% higher head rice yield in the dry season than in the wet season. This apparent effect of the environment on head rice yield is likely attributable to temperature and solar radiation (Deng et al., 2015; Liu et al., 2015), which are higher in the dry season in Senegal at the crop's late reproductive stage. Average percentage grain chalkiness was higher in the wet season in Senegal than in the other trials. This can be explained by the lower daily minimum temperature during the late stages of grain filling. Zhao and Fitzgerald (2013) found a negative relationship between daily minimum temperature and chalkiness at that stage. Together with previous studies, our results for head rice yield and chalkiness indicate that these traits should be carefully evaluated in varietal screening together with weather data, if weather conditions and crop duration vary during the rice growing season.

Compared with head rice and chalkiness, starch properties (apparent amylose content and RVA paste viscosity) of varieties were influenced to a lesser extent by environment. This suggests that data from multilocational trials might be less important for evaluating these traits, and the selection of breeding lines using these traits might be made based on a single trial as their heritability was also high. Between amylose content and RVA paste properties, amylose content was more stable to environment effects. As apparent amylose content is associated with pasting properties of rice, similar variability could have been expected. However, diversity in pasting properties was wider confirming differences in pasting properties of varieties with similar amylose content (Cuevas & Fitzgerald, 2012) and suggesting that RVA properties may be a more precise indication of rice eating quality than amylose content, which is often used to predict rice eating quality, especially texture (Sowbhagya, Ramesh, & Bhattacharya, 1987; Yu, Ma, & Sun, 2009).

### 4.2 Relationships among agronomic and grain quality traits

None of the agronomic traits correlated consistently with any of the grain quality traits across the four trials. This implies that no agronomic trait can be relied on to effectively predict any rice grain quality trait and accentuates the need to integrate the selection for grain quality in the early stages of the varietal development process instead of evaluating grain quality after advanced lines have been selected.

Rice grain chalkiness has often been associated with low head rice yield to the extent that it has been suggested that selecting germplasm that have reduced chalk under multiple environments could be a viable way of enhancing head rice yield and reducing susceptibility to chalk‐mediated breakage under stress conditions on the premise that high chalkiness makes grains fragile and prone to breakage during milling (Liu et al., 2015; Sreenivasulu et al., 2015; Zhou et al., 2015). Results of this study contradicted these assertions as chalkiness did not have consistent significant correlations with head rice yield implying that predicting head rice recovery based on chalkiness may not be reliable in some environments.

Again, contrary to previous reports that rice chalkiness decreases with decreasing grain width (Tan et al., 2000), no consistent correlation was observed between chalkiness and grain dimensions. Grain dimensions have also often been used, particularly in the United States, as a predictor of rice eating quality, and although Mestres, Ribeyre, Pons, Fallet, and Matencio (2011) observed that cooked rice texture did not conform with classical grain ranking based on shape but rather chemical properties such as AAC, the consistent correlation of grain dimensions with AAC as observed in this study lends some credence to the reliability of such classification of eating quality based on grain shape. Zhou et al. (2015) observed that head rice yields were highest in low amylose varieties; this study likewise observed consistent significant negative correlation between head rice yield and AAC, implying that lower average head rice yield recorded in comparison with other studies may be associated with the fact that most varieties in this study were high‐amylose types.

Although similar correlations as reported by Allahgholipour, Ali, Alinia, Nagamine, and Kojima (2006) and Gayin, Manful, and Johnson (2009) were observed between apparent amylose content and pasting properties, our results showed that among varieties with similar apparent amylose contents, there were substantial variations in their pasting properties, as their relationships were weakly significant. Thus, these three traits of breeding lines should be evaluated rather than measuring one trait only and relying on general relationships among them.

### 4.3 Classification of varieties based on paddy yield, head rice yield, and chalkiness across trials

While varieties in Cluster 1 have the potential for high grain quality and good paddy yield, especially in Senegal, those in Cluster 2 have high and stable paddy yield but poor head rice yield. Sahel 108 in Cluster 1 and Jaya in Cluster 2 showed relatively higher paddy yield, not only in Senegal but also in Benin. It is evident from the population of group 3 that most local cultivars do not perform consistently in yield nor grain quality across trials. Most have good performance in one of the three traits in only one environment. This makes a case for the need not only to select varieties with good performance across environments but also to select varieties suitable traits for specific environments. The varieties in group 4 with consistently low chalkiness but poor yields (both paddy and head rice) provide an option for crossing with higher yielding varieties to improve their grain qualities. Varieties such as Sahel 108, IR64, and Jaya could serve as donors for improving both paddy yield and grain quality, and as reference checks in multilocation trials to identify new breeding lines that combine these traits.

## 5 CONCLUSIONS

Current market demands in West Africa require target outputs of rice breeding programs to be aligned toward improved grain quality traits together with higher paddy yield. This study reveals that there exist wide variations in the agronomic and grain quality traits of rice varieties available in West Africa. The apparent diversity in these traits offers the potential to develop new varieties with high paddy yield and grain quality traits to meet consumer requirements. Sahel 108, IR64, and Jaya can be utilized to breed for high yield combined with good grain quality, and serve as references in multilocational trials. Further systematic screening of available rice varieties, including O*ryza glaberrima,* within the region is needed to reveal the diversity that could be exploited in regional breeding programs. Also, given the nature of the effect of the environment on head rice yield and chalkiness, these traits need to be carefully evaluated during varietal screening together with climatic data particularly where climatic conditions and crop durations vary during the rice growing season.
